# Urinary Tract Infection Frequency and Prescription Prophylaxis in Females and Males with Recurrent Urinary Tract Infection

**DOI:** 10.3390/pathogens12020170

**Published:** 2023-01-21

**Authors:** Amber M. Goedken, Kendra Y. Foster, Erika J. Ernst

**Affiliations:** Division of Health Services Research, College of Pharmacy, University of Iowa, Iowa City, IA 52242, USA

**Keywords:** recurrent urinary tract infection, prophylaxis, antibiotic, methenamine, estrogen

## Abstract

Females and males with recurrent urinary tract infections may receive prescription prophylaxis to reduce the infection frequency. Little is known about how prescription prophylaxis differs between patients meeting and exceeding the minimum threshold for recurrent urinary tract infections. The objectives of this study were to estimate the association between infection frequency and receipt of prescription prophylaxis and describe the type of prescription prophylaxis initiated. This observational study used de-identified fully-insured commercial insurance data from the Midwest from 2003–2016 to identify females and males under age 64 with recurrent urinary tract infections. The patients were categorized as having three or more urinary tract infections in twelve months or only two infections in six months. Multiple logistic regression models were used to determine the association between the infection frequency and receipt of prophylaxis. The frequency of the type of prophylaxis initiated was measured. The odds of receiving prophylaxis were greater in the females and males with three or more infections compared to the patients with only two infections. Estrogen prophylaxis was initiated at a higher rate in females aged 45–63 with two infections than the females with three or more infections. Prescription prophylaxis in females and males with recurrent urinary tract infections differs between those meeting and exceeding the minimum frequency threshold.

## 1. Introduction

Recurrent urinary tract infections are burdensome and costly for the affected patients [1,2]. Adults with recurrent urinary tract infection have lower health-related quality of life compared to the general population [3]. Patients with neurological diseases have an elevated risk of urinary tract infections [4]. Urinary tract infections may exacerbate lower urinary tract symptoms (LUTS) already experienced by patients with neurological diseases [5,6,7]. Patients with LUTS have reduced health-related quality of life in the absence of urinary tract infections [8,9], so recurrent urinary tract infection may be especially burdensome for patients with neurological diseases. Females and males of any age can be affected by recurrent urinary tract infections. Prescription medication, either in addition to or in place of behavioral modification and/or nonprescription products, may be used in patients with recurrent urinary tract infections to reduce future infection frequency. The perceived benefits and risks of prescription prophylaxis for a patient and the decision whether to initiate such prophylaxis depend on the patient’s characteristics. Receipt of prescription prophylaxis may be associated with the clinical characteristic of current infection frequency. A common definition of recurrent urinary tract infection is two urinary tract infections in six months or three or more urinary tract infections in twelve months [10]. It is unknown if patients meeting only the minimum threshold for recurrent urinary tract infection as commonly defined (i.e., two infections in six months) have similar odds of receiving prescription prophylaxis compared to patients with infection frequencies above the minimum threshold.

The efficacy of antibiotic prophylaxis in lowering infection risk is well-established for adult females with uncomplicated urinary tract infections [11] but not established for adults with complicated infections. The risks of antibiotic prophylaxis may include gastrointestinal and other side effects, including *C. difficile* [11,12], and development of antimicrobial resistance [12,13]. Patients may initiate nonantibiotic prophylaxis to avoid the risks of antibiotics. Higher-strength methenamine products provide a prescription alternative to antibiotic prophylaxis for females and males, and vaginal estrogen offers a second prescription alternative for postmenopausal females. It is unknown if the type of prescription prophylaxis initiated differs by infection frequency.

The first objective of this study was to estimate the association between infection frequency and the receipt of prescription prophylaxis. The second objective was to describe the type of prescription prophylaxis initiated by patients with two infections in six months compared to patients with three or more infections in twelve months.

## 2. Materials and Methods

### 2.1. Data Source and Study Population

This observational study used de-identified, fully-insured commercial insurance data from the Midwest from 2003–2016. Recurrent urinary tract infection was defined as two infections in six months or three or more infections in twelve months. The patients with infections meeting the definition were identified.

Urinary tract infection was defined as an inpatient stay or outpatient service with a primary diagnosis of urinary tract infection, including both cystitis and pyelonephritis (Table S1). An outpatient service was required to be accompanied by a dispensed antibiotic within five days before or after the service. To identify the unique urinary tract infections, the start and end dates for each infection were estimated. For an infection identified via an inpatient stay, the start date was the admission date, and the end date was 14 days after the admission date. For an infection identified via an outpatient service, the start date was the earlier of the antibiotic dispensing date or the service date, and the end date was 14 days after the antibiotic dispensing date. Any stays or services with a primary diagnosis of urinary tract infection or antibiotics dispensed from the start date through the end date of an infection were considered related to that infection. A subsequent infection could not start until the previous infection had ended, meaning the start date of the subsequent infection had to occur after the end date of the previous infection.

Patient eligibility for the study was based on the first instance the patient met the definition of recurrent urinary tract infection. The two or three infections contributing to a patient first meeting the definition formed the basis for eligibility. The infection causing the patient to meet the definition will henceforth be called the defining infection, and the infection before that will be called the predefining infection. Patients with a gap in enrollment from one year before the predefining infection through one year after the defining infection were excluded. Patients aged 64 and older at the time of the defining infection were excluded to avoid Medicare eligibility during follow-up. Patients under age 2 were excluded, because children under age 2 are distinct from older children regarding urinary tract infections [14]. Patients dispensed ≥28-day supply urinary tract infection prophylaxis antibiotic (Table S2), ≥28-day supply methenamine-containing product, or vaginal estrogen from one year before the predefining infection up until the defining infection were excluded.

### 2.2. Study Measures

The measured patient characteristics included the age at the time of the defining infection, gender, pregnancy, and comorbidities complicating urinary tract infections. A comorbidity was considered present if there was an inpatient stay or outpatient service with that diagnosis in the year before the predefining infection. The year of the defining infection was identified for use in the analyses. The measure of infection frequency distinguished patients with two infections from patients with three or more infections. Among the patients with three or more infections, some met the definition of recurrent urinary tract infection with their second infection and were categorized separately.

Prescription prophylaxis was considered received if ≥28-day supply urinary tract infection prophylaxis antibiotic (Table S2), ≥28-day supply methenamine-containing product, or vaginal estrogen was dispensed during the year after the defining infection. For patients with three or more infections who met the definition of recurrent urinary tract infection with their second infection, part of the year for identifying the receipt of prophylaxis occurred after their third infection, but some of the year occurred between their second and third infections.

A measure specific to the patients receiving prescription prophylaxis was the type of prophylaxis initiated. The type of prophylaxis initiated (methenamine only, estrogen only, or antibiotic) included all the prophylaxis medications dispensed in the seven-day window starting the day the first prophylaxis medication was dispensed. The seven-day window permitted early additions of antibiotics, or switches from nonantibiotic to antibiotic prophylaxis, to be categorized as antibiotic. Less than one week of use is not enough time for nonantibiotic prophylaxis to demonstrate ineffectiveness, so early additions of or switches to antibiotics indicate a desire to use antibiotic prophylaxis at the outset, assuming the switches not due to side effects.

### 2.3. Data Analysis

The frequencies of infections, comorbidities, pregnancy, year, and receipt of prescription prophylaxis were generated for subgroups of the study population by gender and age (females aged 2–7, aged 8–14, aged 15–17, aged 18–24, aged 25–34, aged 35–44, aged 45–54, and aged 55–63, and males aged 2–17 and aged 18–63). The males were divided into only pediatric and adult subgroups because of the relatively small number of males eligible for the study. For those receiving a prescription prophylaxis, the frequencies of the type of prophylaxis initiated were calculated by gender and age. The frequencies of receipt of prescription prophylaxis by infection frequency were determined by gender and age. Multiple logistic regression models of prescription prophylaxis receipt were performed by gender and age. The first set of models included infection frequency and year. The second set of models included infection frequency, year, pregnancy for females of childbearing age, and comorbidities. The frequencies for the type of prophylaxis initiated by the infection frequency were determined by gender and age. The analyses were performed with SAS 9.4 (SAS, Cary, NC, USA).

### 2.4. Ethical Approval

The study was approved by the University of Iowa Institutional Review Board (IRB ID# 201809728).

## 3. Results

### 3.1. Patient Characteristics

**Figure 1:**
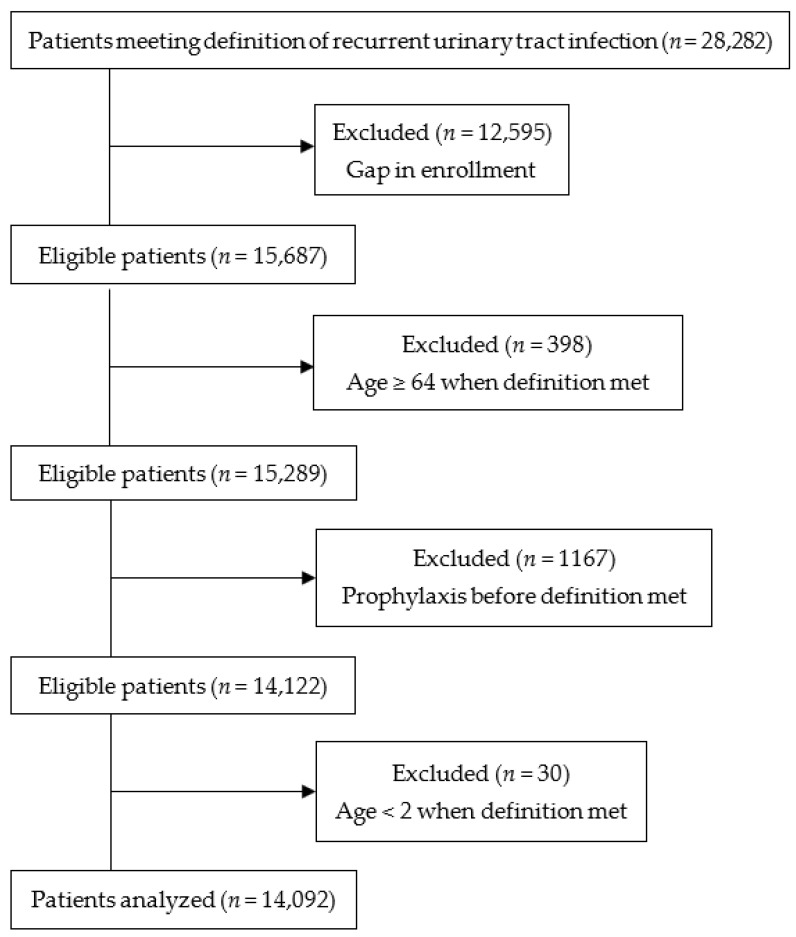
Flow diagram of the study population.

The study included 14,092 patients identified as having recurrent urinary tract infection (Figure 1), of whom 95% were females. Of the females and males with three or more infections, more developed a third infection after meeting the definition of recurrent urinary tract infection than met the definition with their third infection (Table 1). Among the females, the females aged 25–34 had the lowest rate of three or more infections at 36.5%, and females aged 2–7 had the highest rate of three or more infections at 42.0%. Urologic abnormalities, urinary retention, neurogenic bladder, catheterization, advanced kidney disease, and organ transplantation occurred in less than 2% of females, and less than 3% had a neurologic condition (Table S3). The rates of diabetes increased with age, affecting 15.2% of females aged 55–63. Only 59 males with recurrent urinary tract infections were under age 18. The rate of three or more infections among adult males was 28.4%. The rates of urinary tract stones, neurologic conditions, urologic abnormalities, urinary retention, neurogenic bladder, catheterization, advanced kidney disease, and organ transplantation were higher in the adult males than the adult females.

### 3.2. Receipt of Prescription Prophylaxis and Type of Prophylaxis Initiated

The rates of receipt of prescription prophylaxis among the females were lowest in the females aged 15–17, at 9.1%, and highest in the females aged 2–7, at 26.1% (Table 2). In the adult females, the rates of receipt were the highest in females aged 55–63, at 20.7%. The rate of receipt in the adult males was 18.7%.

Of all the females and males initiating antibiotic prophylaxis, only 35 females aged 25–63 initiated antibiotics in combination with methenamine or estrogen. The type of prophylaxis initiated in children was almost always antibiotic prophylaxis in the females and always antibiotic prophylaxis in the males. No female or male children initiated methenamine. No adult females aged 18–24 initiated methenamine, and less than 2% of the females over age 24 who received prophylaxis initiated methenamine only. Methenamine prophylaxis was initiated in 7% of the adult males receiving prophylaxis. Estrogen only prophylaxis was initiated in nearly 40% of the females aged 55–63 and approximately 26% of the females aged 45–54 who received prophylaxis.

### 3.3. Association between Infection Frequency and Receipt of Prescription Prophylaxis

For the females of all ages, the rates of prescription prophylaxis receipt were higher in the females with three or more infections than in the females with two infections (Figure S1). The rates of prophylaxis receipt were also higher in the adult males with three or more infections compared to the males with two infections. Adjusting for year of infection, the odds of receiving prescription prophylaxis were significantly greater for the females of all ages with three or more infections, who developed a third infection after meeting the definition of recurrent urinary tract infection, compared to the females with two infections (Table 3). The odds ratios across the age subgroups ranged from 2.94 to 4.86. The odds of receiving prophylaxis were greater for the females with three or more infections, meeting the definition with their third infection, compared to the females with two infections, though not significantly for females aged 15–17. The odds ratios across the age subgroups ranged from 2.20 to 4.71. The odds of prophylaxis receipt were three-times greater for the adult males with three or more infections compared to the males with two infections. The odds ratios from the second set of models, including infection frequency, year, pregnancy, and comorbidities, were similar to the odds ratios from the more parsimonious first set of models including infection frequency and year, so only the odds ratios from the first set of models are reported.

### 3.4. Type of Prescription Prophylaxis Initiated by Infection Frequency

**Figure 2:**
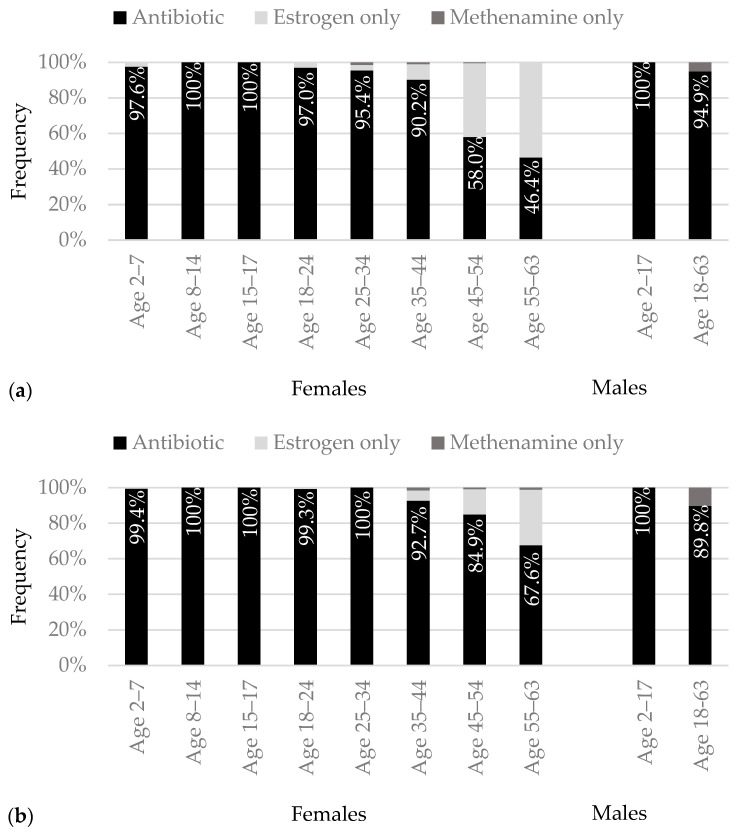

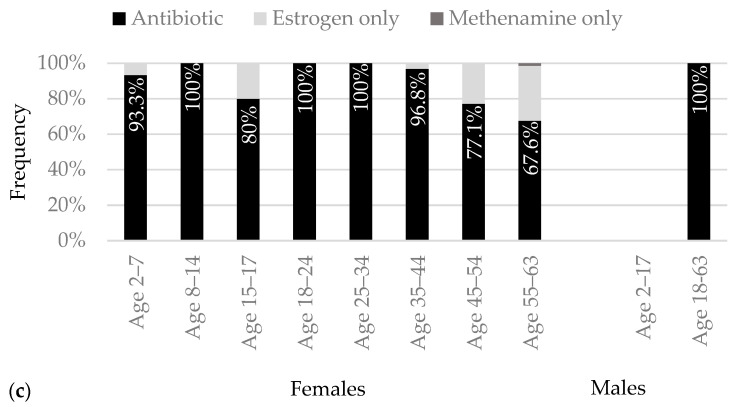
Frequency of initiating each type of prophylaxis by gender and age for (**a**) two infections, (**b**) three or more infections, met definition of recurrent urinary tract infection with second infection, and (**c**) three or more infections, met definition of recurrent urinary tract infection with third infection.

The infrequent initiation of methenamine prevented its inclusion in comparisons between the females with two infections and those with three or more infections. The rates of estrogen-only prophylaxis were higher in the females aged 45–54 and aged 55–63 with two infections compared to those with three or more infections (Figure 2). The infrequent initiation of methenamine prevented comparisons between the males with two infections and three or more infections.

## 4. Discussion

The receipt of prescription prophylaxis in the females and males with recurrent urinary tract infections was associated with the infection frequency. The female children and adults with three or more infections in twelve months had two- to five-times greater odds of receiving prescription prophylaxis compared to the females with two infections in six months. The adult males with three or more infections had three-times greater odds of receiving prophylaxis compared to the males with two infections. For the type of prophylaxis initiated, methenamine initiation was infrequent and limited to the adults. Estrogen-only initiation occurred more frequently in the females aged 45–63 with two infections than with three or more infections.

No previous literature exists for comparison of the direction or magnitude of association between infection frequency and receipt of prescription prophylaxis in recurrent urinary tract infection found in our study. The association between infection frequency and prescription prophylaxis is not a function of the guidelines, as the guidelines for recurrent urinary tract infection do not indicate that prescription prophylaxis should be delayed until after a third infection [15,16]. For the children, premenopausal females, and adult males in our study, the decision to initiate prescription prophylaxis was essentially a decision whether to initiate antibiotic prophylaxis, because methenamine initiation was infrequent. The perceived benefits of antibiotic prophylaxis outweighed the risks for more of the patients with three or more infections than for the patients with two infections. For the postmenopausal females in our study, the decision to initiate prescription prophylaxis was essentially a decision whether to initiate antibiotic or vaginal estrogen prophylaxis. The benefits of prescription prophylaxis outweighed the risks for more of the postmenopausal females with three or more infections than the females with two infections. The females and males who did not receive prescription prophylaxis did not necessarily forgo prophylaxis. They may have used behavioral modification and/or nonprescription products to reduce future infection frequency [2].

Methenamine was initiated infrequently in our study. The emerging evidence for the efficacy of methenamine in preventing infections in adult females could prompt more females to initiate methenamine in the future [17,18]. Because of the infrequent methenamine initiation, comparisons between the females and males with three or more infections and those with two infections, in terms of the type of prophylaxis initiated, could only be made for the postmenopausal females. There was greater willingness to initiate antibiotic prophylaxis, despite the risks, in the females with three or more infections than the females with two infections. The greater willingness may be attributable to a larger evidence base for the efficacy of antibiotic prophylaxis in adult females with three or more infections than in adult females with only two infections [11,19].

The study findings should be considered in the context of a few limitations. Insurance data do not contain laboratory values, patient-reported symptoms, or indications for medications. The episodes labeled as urinary tract infections may have been asymptomatic bacteriuria. The misclassification of episodes as urinary tract infections was minimized by requiring either an inpatient stay or outpatient service with a dispensed antibiotic. The medications labeled as prophylaxis may have been prescribed for a non-urinary tract infection indication. The misclassification of antibiotics as prophylaxis was minimized by limiting them to specific antibiotics with a ≥28-day supply.

## 5. Conclusions

The females and males meeting the minimum frequency threshold for recurrent urinary tract infection are not viewed synonymously with the females and males above the minimum threshold regarding decisions about prophylaxis. Further confirmation with additional populations would be beneficial. Future studies of recurrent urinary tract infection should consider the patients meeting the threshold separately from the patients exceeding the threshold.

## Figures and Tables

**Table 1:** Infection frequency in patients with recurrent urinary tract infection by gender and age.

	*n* (%)									
	Females								Males	
	Age 2–7 (*n* = 1043)	Age 8–14 (*n* = 497)	Age 15–17 (*n* = 749)	Age 18–24 (*n* = 1991)	Age 25–34 (*n* = 1695)	Age 35–44 (*n* = 2007)	Age 45–54 (*n* = 2738)	Age 55–63 (*n* = 2697)	Age 2–17 (*n* = 59)	Age 18–63 (*n* = 616)
Infection frequency										
Two infections	605 58.0%	294 59.2%	459 61.3%	1211 60.8%	1077 63.5%	1267 63.1%	1686 61.6%	1592 59.0%	33 55.9%	441 71.6%
Three or more infections, met definition of recurrent urinary tract infection with second infection	364 34.9%	167 33.6%	238 31.8%	631 31.7%	491 29.0%	567 28.3%	834 30.5%	840 31.2%	23 39.0%	153 24.8%
Three or more infections, met definition of recurrent urinary tract infection with third infection	74 7.1%	36 7.2%	52 6.9%	149 7.5%	127 7.5%	173 8.6%	218 8.0%	265 9.8%	3 5.1%	22 3.6%

**Table 2:** Receipt of prescription prophylaxis and type of prophylaxis initiated by gender and Age.

	*n* (%)									
	Females								Males	
	Age 2–7 (*n* = 1043)	Age 8–14 (*n* = 497)	Age 15–17 (*n* = 749)	Age 18–24 (*n* = 1991)	Age 25–34 (*n* = 1695)	Age 35–44 (*n* = 2007)	Age 45–54 (*n* = 2738)	Age 55–63 (*n* = 2697)	Age 2–17 (*n* = 59)	Age 18–63 (*n* = 616)
Received prescription prophylaxis	272 26.1%	92 18.5%	68 9.1%	237 11.9%	181 10.7%	257 12.8%	461 16.8%	559 20.7%	26 44.1%	115 18.7%
Type of prescription prophylaxis initiated in patients receiving prophylaxis										
Antibiotic	267 98.2%	92 100%	67 98.5%	234 98.7%	178 98.3%	237 92.2%	337 73.1%	334 59.8%	26 100%	107 93.0%
Estrogen only	5 1.8%	--	1 1.5%	3 1.3%	2 1.1%	17 6.6%	121 26.3%	221 39.5%	--	--
Methenamine only	--		--	--	1 0.6%	3 1.2%	3 0.7%	4 0.7%	--	8 7.0%

**Table 3:** Multiple logistic regression models by gender and age: association between infection frequency and receipt of prescription prophylaxis.

	Receipt of Prescription Prophylaxis Odds Ratio (95% CI)									
	Females								Males	
	Age 2–7 (*n* = 1043)	Age 8–14 (*n* = 497)	Age 15–17 (*n* = 749)	Age 18–24 (*n* = 1991)	Age 25–34 (*n* = 1695)	Age 35–44 (*n* = 2007)	Age 45–54 (*n* = 2738)	Age 55–63 (*n* = 2697)	Age 2–17 (*n* = 59)	Age 18–63 (*n* = 616)
Infection frequency										
Two infections	Ref	Ref	Ref	Ref	Ref	Ref	Ref	Ref		Ref
Three or more infections, met definition of recurrent urinary tract infection with second infection	4.86 (3.55, 6.64)	4.58 (2.75, 7.62)	4.48 (2.58, 7.79)	4.75 (3.47, 6.48)	3.84 (2.75, 5.38)	3.19 (2.40, 4.24)	2.94 (2.37, 3.64)	3.34 (2.72, 4.10)		3.31 (2.12, 5.17)
Three or more infections, met definition of recurrent urinary tract infection with third infection	4.29 (2.55, 7.22)	2.72 (1.12, 6.62)	2.20 (0.79, 6.12)	4.71 (2.96, 7.49)	2.74 (1.58, 4.74)	2.49 (1.61, 3.86)	2.26 (1.59, 3.23)	2.58 (1.90, 3.50)		3.15 (1.22, 8.14)

CI: confidence interval; Ref: reference group. Models adjusted for year. Model could not be fitted for males aged 2–17.

## Data Availability

Restrictions apply to the availability of these data.

## Supplementary Materials

The following supporting information can be downloaded at https://www.mdpi.com/article/10.3390/pathogens12020170/s1: Table S1: Codes used to identify urinary tract infections; Table S2: Urinary tract infection prophylaxis antibiotics; Table S3: Characteristics of patients with recurrent urinary tract infection by gender and age; Figure S1: Frequency of receiving prescription prophylaxis by gender and age.
