# Supplementary material for: Urinary Tract Infection Frequency and Prescription Prophylaxis in Females and Males with Recurrent Urinary Tract Infection
Source: Pathogens. 2023 Jan 21;12(2):170. doi: 10.3390/pathogens12020170 (PMC9961979; doi:10.3390/pathogens12020170)
Supplement: Supplementary file 1 [file pathogens-12-00170-s001.zip › pathogens-2163090-supplementary.pdf]

pathogens

Table S1. Codes used to identify urinary tract infections.

International Classification of Diseases, Ninth Revision, Clinical Modification (ICD-9-CM) codes	International Classification of Diseases, Tenth Revision, Clinical Modification (ICD-10-CM) codes
590.10	N10
590.11	N12
590.2	N15.1
590.3	N15.9
590.80	N16
590.81	N28.84
590.9	N28.85
595.0	N28.86
595.9	N30.00
599.0	N30.01
	N30.90
	N30.91
	N39.0

Table S2. Urinary tract infection prophylaxis antibiotics.

**Medication name**
Nitrofurantoin
Sulfamethoxazole/trimethoprim
Trimethoprim
Cephalexin
Cefaclor
Ciprofloxacin
Levofloxacin
Norfloxacin
Ofloxacin
Gatifloxacin
Nalidixic acid
Cinoxacin
Fosfomycin

Table S3. Characteristics of patients with recurrent urinary tract infection by gender and age.

	n (%)									
	Females								Males	
	**Age 2-7 (n = 1043)**	**Age 8-14 (n = 497)**	**Age 15-17 (n = 749)**	**Age 18-24 (n = 1991)**	**Age 25-34 (n = 1695)**	**Age 35-44 (n = 2007)**	**Age 45-54 (n = 2738)**	**Age 55-63 (n = 2697)**	**Age 2-17 (n = 59)**	**Age 18-63 (n = 616)**
Comorbidities										
Organ transplant	--	1 0.2%	--	3 0.2%	1 0.1%	4 0.2%	7 0.3%	9 0.3%	1 1.7%	8 1.3%
Advanced kidney disease	--	--	--	3 0.2%	7 0.4%	6 0.3%	12 0.4%	38 1.4%	3 5.1%	14 2.3%
Catheterization	11 1.1%	1 0.2%	2 0.3%	5 0.3%	12 0.7%	7 0.4%	26 1.0%	25 0.9%	2 3.4%	47 7.6%
Neurogenic bladder	9 0.9%	3 0.6%	2 0.3%	8 0.4%	6 0.4%	9 0.5%	30 1.1%	27 1.0%	17 28.8%	58 9.4%
Urinary retention	2 0.2%	4 0.8%	1 0.1%	2 0.1%	12 0.7%	13 0.7%	30 1.1%	23 0.9%	4 6.8%	78 12.7%
Urologic abnormalities	11 1.1%	7 1.4%	4 0.5%	17 0.9%	23 1.4%	26 1.3%	38 1.4%	46 1.7%	2 3.4%	55 8.9%
Neurologic condition	6 0.6%	5 1.0%	10 1.3%	12 0.6%	17 1.0%	30 1.5%	69 2.5%	60 2.2%	13 22.0%	52 8.4%
Urinary incontinence	92 8.8%	25 5.0%	9 1.2%	9 0.5%	26 1.5%	51 2.5%	125 4.6%	128 4.8%	10 17.0%	21 3.4%
Urinary tract stones	--	--	8 1.1%	30 1.5%	39 2.3%	56 2.8%	63 2.3%	68 2.5%	1 1.7%	58 9.4%
Diabetes	2 0.2%	2 0.4%	9 1.2%	23 1.2%	29 1.7%	89 4.4%	215 7.9%	411 15.2%	2 3.4%	79 12.8%
Pregnancy	--	--	21 2.8%	95 4.8%	378 22.3%	114 5.7%	6 0.2%	4 0.2%	--	--
Year										
2004-2007	354 33.9%	180 36.2%	289 38.6%	525 26.4%	612 36.1%	734 36.6%	917 33.5%	875 32.4%	26 44.1%	217 35.2%
2008-2011	397 38.1%	171 34.4%	283 37.8%	757 38.0%	655 38.6%	745 37.1%	1030 37.6%	949 35.2%	22 37.3%	241 39.1%
2012-2015	292 28.0%	146 29.4%	177 23.6%	709 35.6%	428 25.3%	528 26.3%	791 28.9%	873 32.4%	11 18.6%	158 25.7%

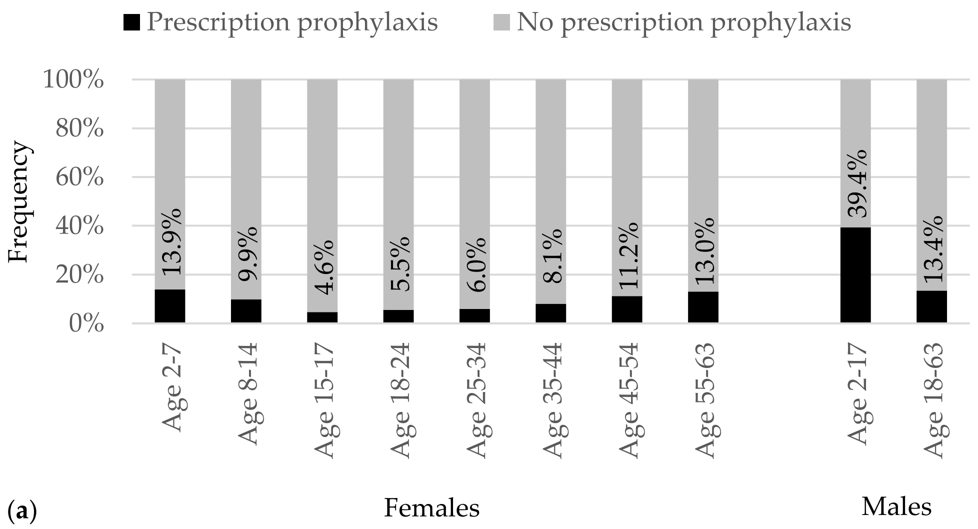
Prescription prophylaxis
No prescription prophylaxis
Frequency
100%
80%
60%
40%
20%
0%
13.9%
9.9%
4.6%
5.5%
6.0%
8.1%
11.2%
13.0%
39.4%
13.4%
Age 2-7
Age 8-14
Age 15-17
Age 18-24
Age 25-34
Age 35-44
Age 45-54
Age 55-63
Age 2-17
Age 18-63
Females
Males

(**a**)

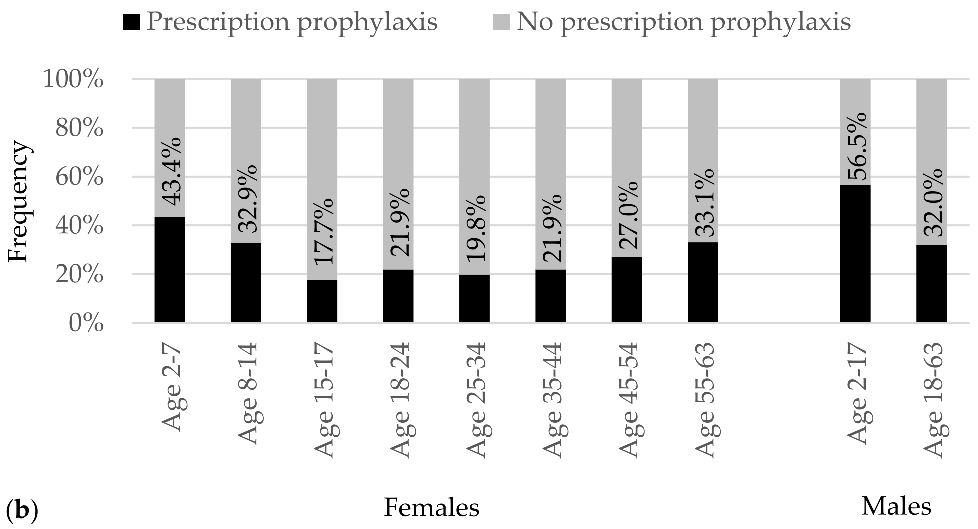
Prescription prophylaxis
No prescription prophylaxis
Frequency
100%
80%
60%
40%
20%
0%
43.4%
32.9%
17.7%
21.9%
19.8%
21.9%
27.0%
33.1%
56.5%
32.0%
Age 2-7
Age 8-14
Age 15-17
Age 18-24
Age 25-34
Age 35-44
Age 45-54
Age 55-63
Age 2-17
Age 18-63
Females
Males

(**b**)

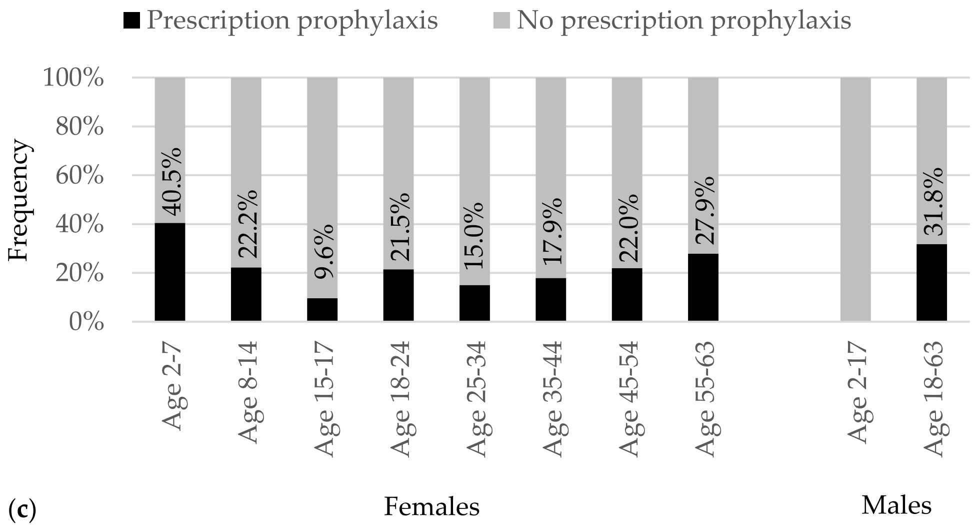
Prescription prophylaxis
No prescription prophylaxis
Frequency
100%
80%
60%
40%
20%
0%
40.5%
22.2%
9.6%
21.5%
15.0%
17.9%
22.0%
27.9%
31.8%
Age 2-7
Age 8-14
Age 15-17
Age 18-24
Age 25-34
Age 35-44
Age 45-54
Age 55-63
Age 2-17
Age 18-63
Females
Males

(**c**)

Figure S1. Frequency of receiving prescription prophylaxis by gender and age for (**a**) two infections, (**b**) three or more infections, met definition of recurrent urinary tract infection with second infection, and (**c**) three or more infections, met definition of recurrent urinary tract infection with third infection.
